# Effect of Interaction between Chromium(VI) with 17β-Estradiol and Its Metabolites on Breast Cancer Cell Lines MCF-7/WT and MDA-MB-175-VII: Preliminary Study

**DOI:** 10.3390/molecules28062752

**Published:** 2023-03-18

**Authors:** Ewa Sawicka, Julita Kulbacka, Małgorzata Drąg-Zalesińska, Arkadiusz Woźniak, Agnieszka Piwowar

**Affiliations:** 1 Department of Toxicology, Faculty of Pharmacy, Wroclaw Medical University, Borowska 211, 50-556 Wroclaw, Poland; 2 Department of Molecular and Cellular Biology, Faculty of Pharmacy, Wroclaw Medical University, Borowska 211A, 50-556 Wroclaw, Poland; 3 Department of Immunology, State Research Institute Centre for Innovative Medicine, Santariškių 5, 08410 Vilnius, Lithuania; 4 Division of Histology and Embrylogy, Department of Human Morphology and Embryology, Faculty of Medicine, Wroclaw Medical University, T. Chałubińskiego 6a, 50-368 Wroclaw, Poland; 5 Students’ Scientific Society at the Department of Toxicology, Faculty of Pharmacy, Wroclaw Medical University, 50-556 Wroclaw, Poland

**Keywords:** 17β estradiol, 2-methoxyestradiol, 4-hydroxyestradiol, 16α-hydroxyestrone, metalloestrogens, Cr(VI), MCF-7/WT, MDA-MB-175-VII cytotoxicity, genotoxicity, SOD1 expression

## Abstract

The number of factors initiating and stimulating the progression of breast cancer are constantly increasing. Estrogens are a risk factor for breast adenocarcinoma, the toxicity of which increases as a result of metabolism and interaction with other factors. Due to the presence of environmental exposure to estrogens and metalloestrogens, we investigated how interactions between estrogens and toxic chromium(VI)[Cr(VI)] affect breast cancer lines and investigated whether estrogens play a protective role. The aim of the study was to investigate the effect of 17β-estradiol and its metabolites: 2-methoxyestradiol (2-MeOE2), 4-hydroxyestradiol (4-OHE2), and 16α-hydroxyestrone (16α-OHE1) in exposure to Cr(VI) on cell viability and DNA cell damage. Two estrogen-dependent breast cancer cell lines, MCF 7/WT and MDA-MB-175-VII, were examined. In addition, the expression of Cu-Zn superoxide dismutase (SOD1) was determined immunocytochemically to elucidate the mechanism of oxidative stress. The effects of single substances and their mixtures were tested in the model of simultaneous and 7-day estrogen pre-incubation. As a result, the viability of MCF-7 and MDA-MB-175-VII cells is lowered most by Cr(VI) and least by 17β-E2. In the combined action of estrogens and metalloestrogens, we observed a protective effect mainly of 17β-E2 against Cr(VI)-induced cytotoxicity. The highest expression of SOD1 was found in MCF-7/WT cells exposed to 17β-E2. Moreover, high apoptosis was caused by both Cr(VI) itself and its interaction with 4-OHE2 and 2-MeOE2. The direction and dynamics of changes in viability are consistent for both lines.

## 1. Introduction

Chromium, a transition metal with the most stable hexavalent [Cr(VI)] and trivalent [Cr(III)] forms, is widely distributed in the environment. High levels of exposure to Cr(VI) are mainly connected with industry (chromate manufacturing or chrome plating). Cr(VI) has carcinogenic and mutagenic effects and is considered more toxic than Cr(III) due to its high solubility and mobility. The hexavalent form was recognized by the International Agency for Research on Cancer (IARC) as a carcinogen with a proven carcinogenic effect on humans (class I) [1,2,3]. Many types of structural genetic lesions stimulated by Cr(VI) have been observed in vivo and in vitro, including inter-strand cross-links, DNA protein cross-links, strand breaks, and the creation of Cr-DNA adducts [4]. 

Cr(VI) is a metalloestrogen responsible for many disorders in humans and interacts with estrogen receptors. It generates reactive oxygen species, translocates through biological membranes, and is subsequently reduced intracellularly to Cr(V), Cr(IV), and Cr(III) with simultaneous reactive oxygen species (ROS) generation. It also affects the activity of antioxidant enzymes [5]. Because exposure to Cr(VI) is widespread, the examination of its role in the etiology and development of hormone-dependent cancers, such as breast cancer, may have significant implications for disease prevention. Metalloestrogens induce estrogen-dependent breast cancer cell proliferation and activate estrogen receptor α (ERα), which has been proven in vitro [6].

One of the characteristics of most metalloestrogens is their long biological half-life; hence, their accumulation in the organism, e.g., in mammary gland tissue, has very adverse effects [7,8]. The interaction of estrogens with carcinogenic factors (e.g., chromium) requires special attention; since estrogen displays carcinogenic activity, the synergistic effect with carcinogenic chromium(VI) could be hazardous [9,10]. However, the dualistic role of estrogens should be emphasized. Data from the literature indicate that, in addition to the unfavorable effects of estrogens, they are also observed to have a positive influence in the treatment of breast cancer. Estradiol used in high doses in additive therapy has been shown to induce the regression of breast cancer cells expressing estrogen receptors, and this is the case in postmenopausal women [11]. The use of 2-MeOE2 also has a positive effect on the treatment of breast cancer. The mechanism of action of this metabolite is independent of estrogen receptors, which means that the compound acts on cells that do not express these receptors. The mechanism of 2-MeOE2’s anticancer action functions by damaging the cytoskeleton of cancer cells [12]. In addition, many scientists have studied the protective effect of estrogens, e.g., on bones, which seems to be caused by their ability to attenuate oxidative stress [13]. Moreover, 17β-E2 inhibits quickly, and in the presence of oxidative stress, the osteocyte undergoes apoptosis, and osteoclastogenic factors are activated [14]. The above data indicate the protective properties of estrogens, hence the interest in these properties in breast cancer cell lines. According to the IARC, breast cancer is the second most common cancer in the world and the second cause of cancer deaths in women, just after lung cancer [15]. The incidence of breast cancer, with about 1,700,000 new cases each year, is alarming and requires research in this direction [16]. Estimated data indicate that by 2025, the highest increase in morbidity will appear mainly in postmenopausal women aged 50–69. This may be caused by the constantly increasing number of agents, predominantly exogenous, present in the environment, including Cr(VI) [17,18].

Due to the fact that no studies have examined the role of the interaction between estrogens (17β-estradiol and its metabolites) with Cr(VI) on breast cancer, we became interested in this topic. There has been increasing interest in the role of estrogen metabolites that may affect the risk of breast cancer. The metabolism of estrogens may cause DNA damage by forming mutagenic DNA adducts or free radicals. Two main pathways are present for estrogen metabolism in humans: hydroxylation of the A-ring (2- or 4-hydroxy derivatives) or hydroxylation of the D-ring (at 16α-position) [19]. While 2-hydroxyestradiol is preferentially converted to 2-methoxyestradiol (2 MeOE2), 4-hydroxyestradiol (4-OHE2) is readily oxidized to 3,4 quinone, a genotoxic metabolite. While 2 MeOE2 is characterized as having antiangiogenic, proapoptotic, and antiproliferative properties [20,21], 4-OHE2 and C-16α hydroxylated estrone (16α-OHE1) have stronger estrogenic activity than 17β-estradiol or 2 MeOE2 [22].

Due to the considerable role of DNA modification in the process of carcinogenesis, as well as the small amount of the literature data on the genotoxic effect of estrogens and metalloestrogens, the purpose of this study was to assess the effect of interactions of the metalloestrogen Cr(VI) with 17β-estradiol (17β-E2) and its metabolites 4-OHE2, 16α-OHE1, and 2-MeOE2 on cytotoxicity and DNA damage in vitro in the estrogen-dependent cell lines MCF-7/WT and MDA-MB-175-VII. The genotoxicity of these compounds was checked using the comet method, and cytotoxicity was checked by an MTT test. Since the free radical mechanism is involved in the transformation of chromium(VI) and estrogens, the influence of the tested compounds on the expression of the SOD1 enzyme was assessed by immunocytochemical staining. The examination of the influence of single substances and their mixtures in different combinations (simultaneous and 7-day pre-incubation) on the cell line in concentrations plausibly present in the environment was of particular interest. The important goal of this study was to identify the potential protective role of 17β–E2 and its metabolites under conditions of exposure of breast cancer cells to carcinogenic Cr(VI).

## 2. Results

### 2.1. Viability Assay of Single Compounds (First Stage, First Model)

**Figure 1:**
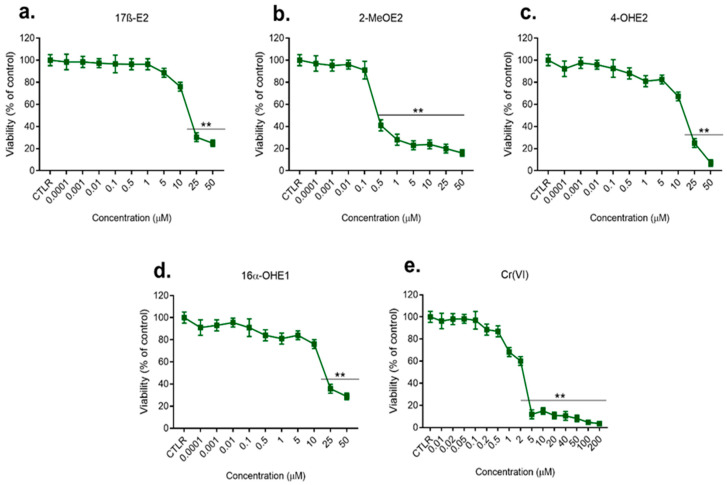
Viability of MCF-7/WT cell line after 72 h exposure to 17β-E2 (**a**) and its metabolites: 2-MeOE2 (**b**), 4-OHE2 (**c**), and 16α-OHE1 (**d**) in concentrations 0.1 nM–50 µM, and to Cr(VI) (**e**) in concentrations 0.01–200 µM, determined by MTT test; significance in comparison to control—CTLR, *p* < 0.01 (**).

**Figure 2:**
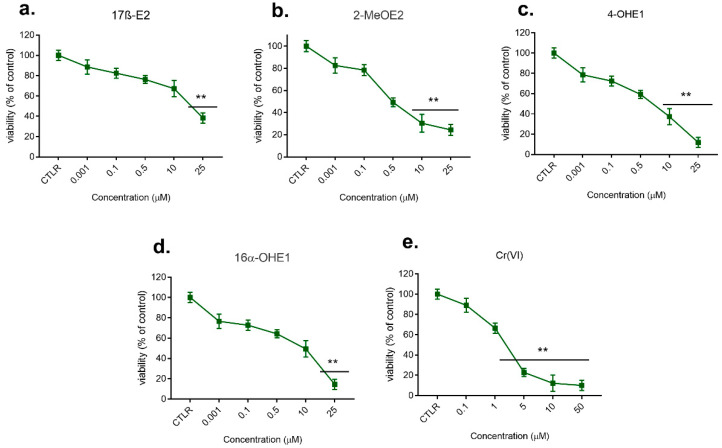
Viability of MDA-MB-175-VII cell line after 72 h exposure to 17β-E2 (**a**) and its metabolites: 2-MeOE2 (**b**), 4-OHE2 (**c**), and 16α-OHE1 (**d**) in concentrations 0.1 nM–25 µM, and to Cr(VI) (**e**) in concentrations 0.1–50µM, determined by MTT test; significance in comparison to control—CTLR, *p* < 0.01 (**).

The results of the first stage of the study, including the effect of the single actions of estrogens, their metabolites, and Cr(VI) on the viability of the cells, determined by the MTT test after 72 h incubation with compounds and conducted for a broad spectrum of used concentrations (0.1 nM–200 μM), are shown in Figure 1 and Figure 2.

#### 2.1.1. Effect of 17β-E2, Its Metabolites, and Cr(VI) on MCF-7/WT Viability

As shown in Figure 1, 17β-E2 at a concentration of 10μM caused a statistically significant decrease in MCF-7/WT viability compared to the control (*p* < 0.05). Higher concentrations (25–50 μM) caused an even more statistically significant decrease in cell viability (30–25%) (*p* < 0.01); 2-MeOE2 in doses of 0.5–50 μM caused inhibition of viability of 60% and 28%, respectively, and even up to 16% at the highest concentration. In the concentration range of 0.1 nM–10 μM, 4-OHE2 inhibited viability insignificantly. A further increase in its concentration (25 μM–50 μM) resulted in a significant reduction of viability—even to 5%. In concentrations of 0.1 nM–10μM, 16α-OHE1 had an insignificant effect. Increasing its dose (25 μM to 50 μM) lowered viability in a statistically significant way to 28% (*p* < 0.01). Cr(VI) (0.01–0.5 μM) showed weak activity in relation to MCF-7/WT cells. Exposure to 1 μM and 2 μM caused a decrease in cell viability to 66% and 48%, respectively. Doses of 5–200 μM caused a very sharp decrease in viability, even to 5%, which indicates the highly cytotoxic properties of Cr(VI) (*p* < 0.001).

#### 2.1.2. Effect of 17β-E2, Its Metabolites, and Cr(VI) on MDA-MB-175-VII Viability

As shown in Figure 2, 17β-E2 at concentrations of 10 μM and 25 μM caused a statistically significant decrease in MDA-MB-175-VII cell line viability compared to control (*p* < 0.01). In doses of 0.5 μM and 50 μM, 2-MeOE2 caused inhibition of viability of 50% and 24%, respectively. In the concentration range of 10μM-25 μM, 4-OHE2 inhibited viability significantly (*p* < 0.01), with a reduction of MDA-MB-175-VII viability up to 8%; 16α-OHE1 showed a very similar effect to 4-OHE2, lowering cell viability even to 10% (*p* < 0.01). Cr(VI) used in doses 0.1–50 μM indicated a differentiated effect. Weak activity in relation to MDA-MB-175-VII cells was noted for 0.1–1.0 μM of Cr(VI). Exposure to concentrations of 5 μM and 50 μM of metalloestrogen decreased cell viability to 20% and 8%, respectively (*p* < 0.01).

### 2.2. Viability Assay of Combined Action of Estrogens with Cr(VI) (First Stage, Second Model)

Based on an analysis of the effect of single compounds, the concentrations listed below were selected to evaluate the combined effect of estrogens and Cr(VI). Additionally, we chose to limit the criteria for selecting concentrations for further experiments to 1 nM at the lowest for all tested estrogens, which is comparable to the physiological concentrations of estrogens present in the organism. Meanwhile, the highest concentration of these compounds was accompanied by the highest inhibition of viability.

#### 2.2.1. Effect of Combined Action of 17β-E2 with Cr(VI) on MCF-7/WT Viability

**Figure 3:**
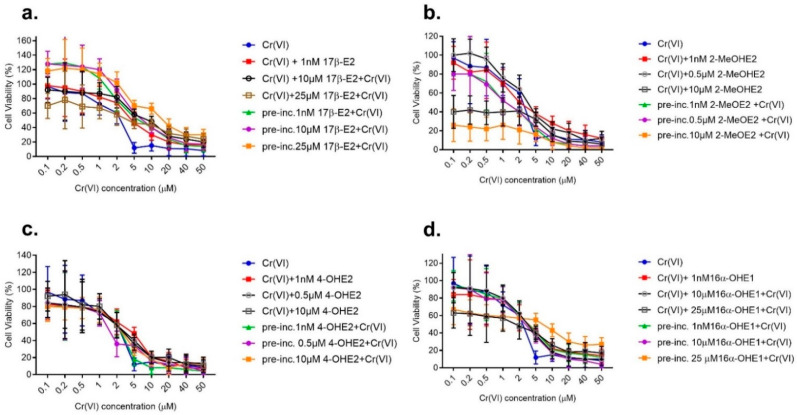
Viability of MCF-7/WT cell line after combined exposure to 17β-E2 and Cr(VI) determined by an MTT test in simultaneous action and in 7-day pre-incubation with 17β-E2 in comparison to Cr(VI) alone (**a**), to Cr(VI) and 2-MeOE2 (**b**), to Cr(VI) and 4-OHE2, and (**c**) to Cr(VI) and 16α-OHE1 determined by an MTT test in simultaneous action and in 7-day pre-incubation with 16α-OHE1 in comparison to Cr(VI) alone (**d**).

Results of the combined action of Cr(VI) (0.1–50 μM) with 17β-E2: 1.0 nM, 10 and 25 μM, in their simultaneous action and in pre-incubation with 17β–E2 are shown in Figure 3a.

Results of the combined action of Cr(VI) (0.1–50 μM) with 17β-E2: 1.0 nM, 10 and 25 μM, in their simultaneous action and in pre-incubation with 17β-E2 are shown in Figure 3a. Concerning simultaneous interaction, regardless of the applied 17β-E2 concentration, there was a visible trend for a reduction in cell line viability, especially in the low range of Cr(VI) concentrations (0.1–1 μM), compared to cells exposed to Cr(VI) alone (mainly for 25 μM 17β-E2). This is different from the test with pre-incubation with estrogen. Cell viability after interaction is close to 100% (mainly for 17β-E2 1 nM) (Figure 3a). In the higher range of Cr(VI) concentrations—above 2 μM (17β-E2: 1 nM and 10 μM) or from 5 μM Cr(VI) and 25 μM 17β-E2—an inverse relationship was observed. In the case of exposure to Cr(VI) preceded by pre-incubation with 17β-E2, an increase in MCF-7/WT viability was noted.

#### 2.2.2. Effect of Combined Action of 2-MeOE2 with Cr(VI) on MCF-7/WT Viability

Analyzing the combined action of 2-MeOE2 with Cr(VI), it was found that in the case of co-exposure to 2-MeOE2 (10 μM) the cell viability was reduced for the low Cr(VI) concentration (Figure 3b). The results of the study show that low concentrations of 2-MeOE2 (especially 1.0 nM) do not affect the inhibition of MCF-7/WT viability in exposure to Cr(VI), in contrast to the higher dose (10.0 μM), for which the changes are very noticeable. In the case of examination of the effects of exposure to Cr(VI) preceded by 7-day pre-incubation of 2- MeOE2, an inhibition of viability compared to the effect caused by the single action of Cr(VI) was noted. A particular decrease in cell viability was observed for 2-MeOE2 (10.0 μM) and in the low dose range of Cr(VI) at 0.1–5.0 μM. The viability is lower than in the case of their simultaneous action (Figure 3b).

#### 2.2.3. Effect of Combined Action of 4-OHE2 with Cr(VI) on MCF-7/WT Viability

Results showed that 4-OHE2 in combined action with Cr(VI) inhibited cell viability but less than the interaction with Cr(VI)-2-MeOE2 and only in certain concentration ranges. The use of 4-OHE2 (1 nM) caused an increase in viability in the presence of Cr(VI) (2–10 μM). On exposure to 10 μM 4-OHE2 and up to 1 μM Cr(VI), the addition of this metabolite caused a slight decrease in viability. Pre-incubation with 10.0 μM 4-OHE2 caused a more visible reduction in cell vitality but only in the low range of Cr(VI) doses at 0.1–0.5 μM (Figure 3c).

#### 2.2.4. Effect of Combined Action of 16α-OHE1 with Cr(VI) on MCF-7/WT Viability

Both at 1 nM and 10 μM, 16α-OHE1 did not cause changes in viability compared to Cr(VI) alone (Figure 3d). The effects of simultaneous action coincide at the highest metabolite concentration (25 μM). In a concentration of 25 μM mixed with up to 2 μM of Cr(VI), 16α-OHE1 causes a decrease in MCF-7/WT viability, but for chromium at 5.0 and 10 μM it has the opposite effect, causing increased cell viability; 16α-OHE1 (25 μM) caused a kind of protective effect against the cytotoxic action of Cr(VI), which was particularly visible at higher concentrations of Cr(VI) (at 5 μM), when the viability increased to approximately 20–30%.

In each experiment that studied viability, the combined effect, 50% inhibition of cell growth (IC50 values), was also calculated. The results are presented in Table 1, Table 2, Table 3 and Table 4.

#### 2.2.5. Effect of Combined Action of 17β-E2 with Cr(VI) on MDA-MB-175-VII Viability

**Figure 4:**
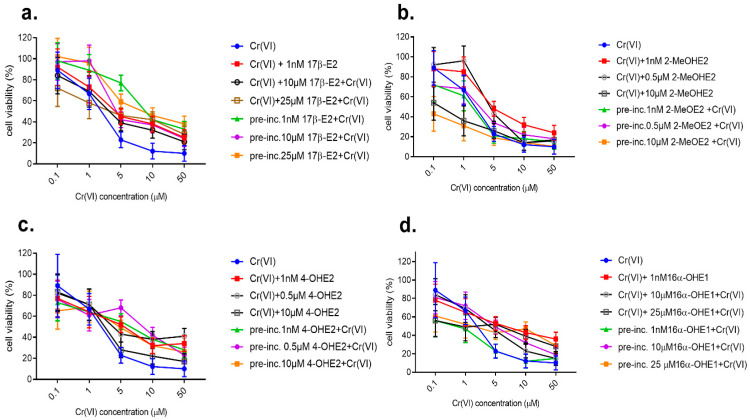
Viability of MDA-MB-175-VII cell line after combined exposure to 17β-E2 and Cr(VI) determined by an MTT test in simultaneous action and in 7-day pre-incubation with 17β-E2 in comparison to Cr(VI) alone (**a**) to Cr(VI) and 2-MeOE2, (**b**) to Cr(VI) and 4-OHE2, and (**c**) to Cr(VI) and 16α-OHE1 determined by an MTT test in simultaneous action and in 7-day pre-incubation with 16α-OHE1 in comparison to Cr(VI) alone (**d**).

In simultaneous interaction with MDA-MB-175-VII, the combined effect of compounds was very similar on both estrogen-dependent cell lines, regardless of the applied 17β-E2 concentration. There was a visible trend for reduction in cell line viability as the dose of the compound was increased. When pre-incubating with 17β-E2 in various doses, its protective effect toward Cr(VI) has been demonstrated, compared to its combined effect with Cr(VI) (Figure 4a).

#### 2.2.6. Effect of Combined Action of 17β-E2 Metabolites with Cr(VI) on MDA-MB-175-VII Viability

When discussing the combined action of 2-MeOE2 with Cr(VI), the trend of changes is different than for 17β-E2 but is very similar to the effect on the MCF-7/MT line. MDA-MB-175-VII viability is higher after interaction than in pre-incubation with 2-MeOE2 (Figure 4b). Results of the combined action of Cr(VI) (from 0.1 μM to 50 μM) with 4-OHE2 at 1 nM and at 0.5 and 10 μM, in their simultaneous action and pre-incubation with this steroid on MDA-MB-175-VII, are presented in Figure 4c and showed 4-OHE2 to inhibit cell viability. A more significant protective effect was demonstrated in the pre-incubation model with this metabolite. On exposure to 10 μM 4-OHE2 and up to 1 μM Cr(VI), the addition of this metabolite caused a slight decrease in MDA-MB-175-VII viability. Results of the combined action of Cr(VI) (at concentrations of 0.1–50 μM) with 16α OHE 1 at 1 nM and 10 and 25 μM, in their simultaneous action and pre-incubation with this steroid, are shown in Figure 4d. Viability changes were not as variable as with 17β-E2 and 2-MeOE2. For the cumulative effect of 16α OHE1, the trend of changes was similar, with a predominance of reduced viability for pre-incubation with this metabolite. The combined effect of estrogen-Cr(VI) showed a similar effect on both tested breast cancer lines. The direction and dynamics of changes are consistent for both lines. Therefore, the further part of the research was performed on one line of MCF-7/WT, especially as both lines belong to estrogen-dependent lines of breast cancer.

### 2.3. Genotoxicity of Single Compounds (Second Stage, First Model: Single Action) Genotoxicity of 17β-E2 and Its Metabolites

**Figure 5:**
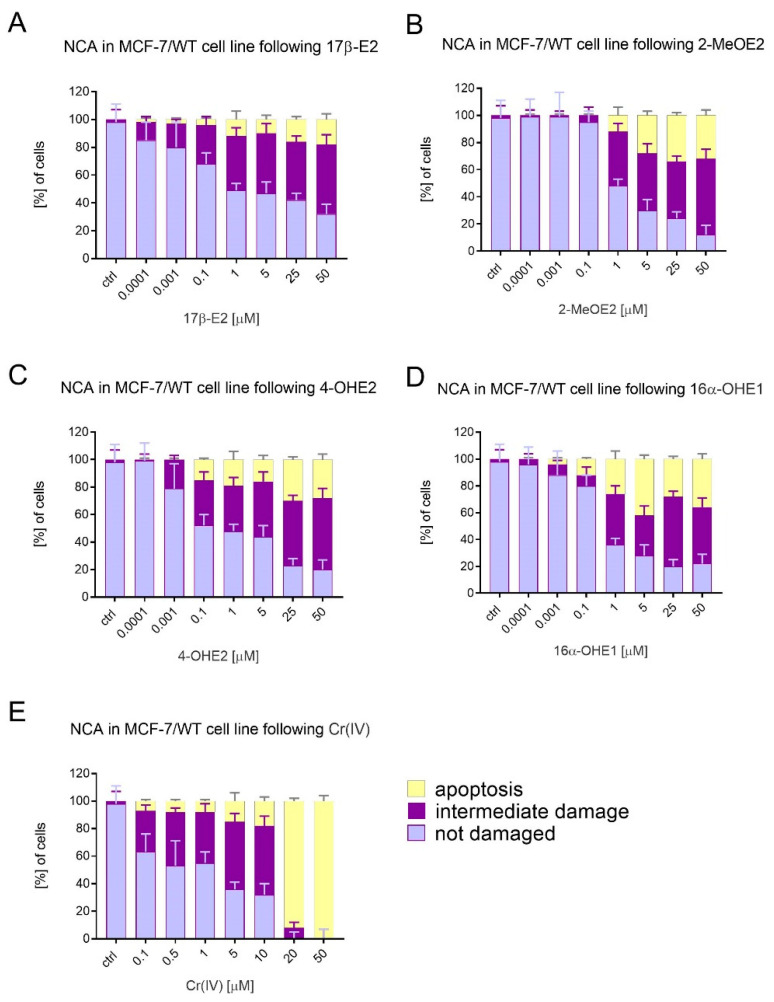
Neutral comet assay carried out after induction with individual estrogens and Cr(VI) alone in comparison to control. NCA detected both the intermediate damage as well as apoptosis and undamaged cells. Estrogens were tested at seven concentrations: 0.1 nM, 1.0 nM; 0.1 μM, 1.0 μM, 5.0 μM, 25 μM, and 50 μM; Cr(VI) was tested in seven concentrations: 0.1 μM, 0.5 μM, 1.0 μM, 5.0 μM, 10 μM, 20 μM, and 50 μM. (**A**) NCA for 17β-E2, (**B**) NCA for 2-MeOE2, (**C**) NCA for 4-OHE2, (**D**) NCA for 16α-OHE1, and (E)) NCA for Cr(VI).

In Figure 5A–D we presented the results obtained from the neutral comet assay carried out after the exposure of MCF-7/WT cells to estrogens and Cr(VI). Concentrations were selected based on viability testing. Estrogens were tested at seven concentrations of 0.1 and 1.0 nM and 0.1, 1.0, 5.0, 25, and 50 μM; Cr(VI) was tested in seven concentrations of 0.1, 0.5, 1.0, 5.0, 10, 20, and 50 μM. The conducted neutral comet assay allowed us to detect both the intermediate damage and apoptosis compared to undamaged cells. At cells’ exposure to 17β-E2 (1 nM-0.1 µM), weak damage predominated in 13–28% of cells. At micromolar concentrations, i.e., 1, 5, 25, and 50 µM, 17β-E2 caused dose-dependent apoptosis of cells at 12, 10, 16, and 18%, respectively. At the highest dose of 17β-E2 (50 μM), the percentage of intact cells was the smallest, at 32% (Figure 5A).

After exposure to 2-MeOE2 in the highest doses, a larger number of cells had undergone apoptosis or showed signs of damage compared to 17β-E2. In the concentration range of 1, 5, 25, and 50 μM, we observed apoptosis at 12, 28, 34, and 32%, respectively. Additionally, the percentage of intermediate damage was observable, and amounted to 40, 42, 42, and 56% for the same concentration range, respectively. The number of undamaged cells decreased statistically as compared to control (Figure 5B). Comparing the effect of both 4-OHE2 and 16α-OHE1, we noted that a visible genotoxic effect on cells started occurring at similar doses of 0.1 µM, at 15% and 12%, respectively. However, when increasing the dose of 16α-OHE1, we noted greater DNA damage, manifested through higher apoptosis, at 1 µM (26%), 5 µM (32%), 25 µM (28%), and 50µM (36%), compared to apoptosis at the same concentrations of 4-OHE2: 19, 16, 30, and 28%, respectively. In contrast, the percentage of intermediately damaged cells was slightly higher for 50 µM of 4-OHE2, reaching 52%, compared with the same dose of 16α-OHE1 (42%). The number of undamaged cells decreased statistically to control (Figure 5C,D).

#### Genotoxicity of Cr(VI)

The genotoxicity detected by the neutral comet test was also observed in cells exposed to Cr(VI), used in seven concentrations: 0.1, 0.5, 1.0, 5.0, 10, 20, and 50 μM; in its highest dose (50 µM), it caused apoptosis in 100% of cells. High DNA damage (apoptosis), with a value of 92%, manifested through apoptosis, has also been demonstrated for a 20 µM Cr(VI) dose. At exposure to Cr(VI) in concentrations from 0.1 to 10 µM, apoptosis ranged between 7 and 18%. For these concentrations, the percentage of cells with intermediate damage was 30–50% (*p* < 0.01) (Figure 5E).

**Figure 6:**
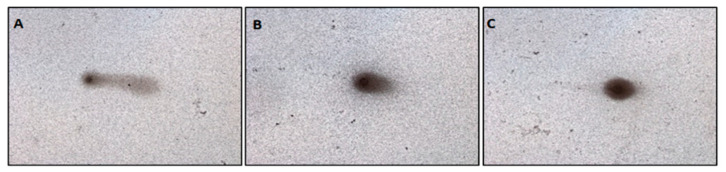
Examples of the microscopic pattern of apoptotic comets. (**A**) Apoptosis after Cr(VI) in concentration 50 μM; (**B**) Intermediate damage after 17β-E2 in concentration 1.0 μM; (**C**) No damage after 2-MeOE2 in concentration 1.0 nM (magnification scale 100×).

An example of the microscopic pattern of apoptotic comets is shown in Figure 6.

### 2.4. Genotoxicity of Combined Action of Estrogens with Cr(VI) (Second Stage, Second Model)

**Figure 7:**
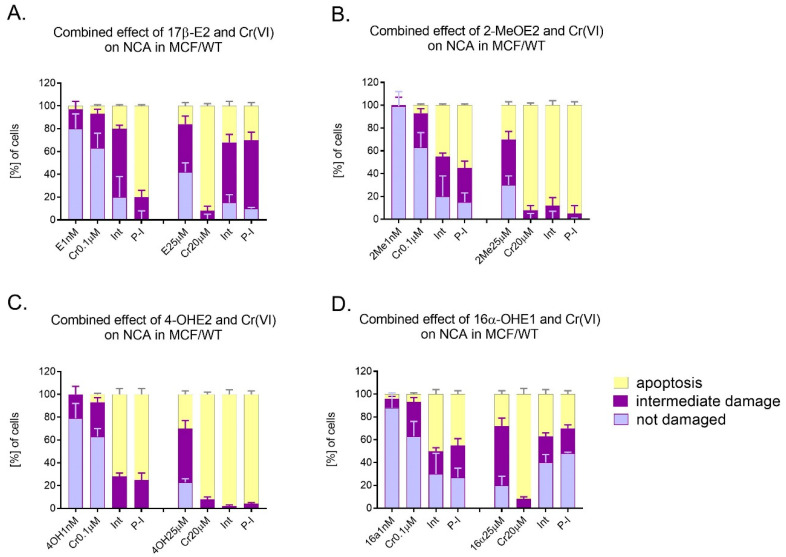
Neutral comet assay carried out after collective action of estrogens in low concentration (1.0 nM) and high concentration (25 µM) with Cr(VI) in low concentration (0.1 µM) and high concentration (20 µM) in relation to MCF-7/WT cells in simultaneous interaction (Int) and pre-incubation with estrogen (P-I). NCA detected both the intermediate damage as well as apoptosis and undamaged cells, (**A**) NCA for 17β-E2 and Cr(VI), (**B**) NCA for 2-MeOE2 and Cr(VI), (**C**) NCA for 4-OHE2 and Cr(VI), and (**D**) NCA for 16α-OHE1 and Cr(VI).

In Figure 7A–D we presented the results of neutral comet assay carried out after the combined action of estrogens with Cr(VI) in relation to MCF-7/WT cells (first and second variant). Two doses of examined compounds were selected for these experiments: a low, nanomolar dose of 17β-E2 (1 nM) and a high dose (25 μM). Similarly, for Cr(VI), a low dose of 0.1 μM and a high dose of 20 μM were used. The combination of 1 nM of 17β-E2 and 0.1 μM of Cr(VI) caused an interaction characterized by large percentage of intermediate damage and 20% apoptosis. After 7-day-long pre-incubation with 17β-E2, high apoptosis (80%) was observed. In turn, the combination of higher concentrations of compounds such as 25 μM of 17β-E2 and 20 μM of Cr(VI), in the case of both simultaneous action and pre-incubation, caused lower DNA damage, estimated as 32% and 30% apoptosis, than after Cr(VI) alone at 92% (Figure 7A). Observing the effect of the mixture of 2-MeOE2 and Cr(VI) at low concentrations (1 nM; 0.1 µM, respectively), high cell genotoxicity was identified in this study. Its level was, respectively, 45% for simultaneous action and 55% for pre-incubation. Neither interaction nor pre-incubation with metabolite reduced the cell damage caused by Cr(VI). The percentage of apoptosis was 88% and 95%, respectively. Thus, almost 100% of the cells showed almost complete DNA damage (apoptosis) (Figure 7B). While the effect of 4-OHE2 (1 nM) or Cr(VI) (1 µM) alone caused low apoptosis of MCF-7 cells, their combined action exerted greater apoptosis, both in the first variant (interaction) and in pre-incubation, achieving 72% and 75% of damage, respectively. Similarly, the combination of 4-OHE2 (25 µM) with Cr(VI) (20 µM) increased the percentage of apoptotic cells to 98% and 96% in interaction and pre-incubation, respectively (Figure 7C). The combination of 16α-OHE1 with Cr(VI) showed a different effect than 4-OHE2, especially in higher doses. The effect of 16α-OHE1 (1nM) or Cr(VI) (1 µM) alone caused low apoptosis (4–7%). However, their combination—simultaneous and pre-incubation with 16α-OHE1—demonstrated higher apoptosis (50% and 45%, respectively), indicating an action similar to a synergistic effect. In contrast to 4-OHE2, the influence of the combination of higher doses of compounds 16α-OHE1 (25 µM) and Cr(VI) (20 µM) exerted apoptosis ranging from 27% to 30% (for simultaneous action and pre-incubation, respectively). Apoptosis in MCF-7/WT caused by a high dose of Cr(VI) was reduced by 16α-OHE1. It is also characteristic that the percentage of undamaged cells after the application of high doses of combined compounds was relatively high (40–48%) for both interaction and pre-incubation (Figure 7D). Mean values for apoptosis, indirect damage, and undamaged cells (neutral comet assay) are shown in the Supplementary Material in Tables S1–S3.

### 2.5. SOD1 Estimation: Immunocytochemical Staining

**Figure 8:**
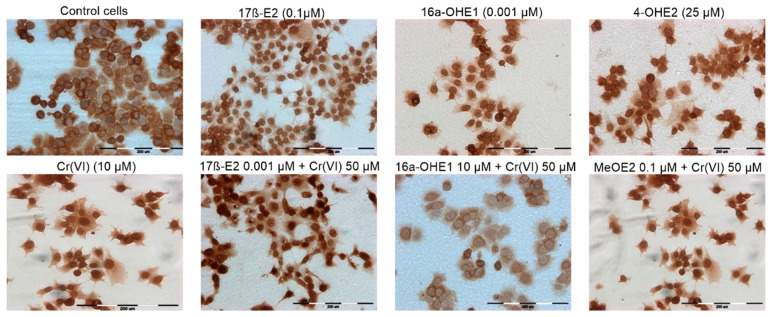
Immunoassayed reaction: exemplary expression results of SOD1 in MCF-7/WT exposed to the single compounds and after exposure to the combined effects of estrogens and Cr(VI).

In the following Table 5, Table 6, Table 7, Table 8 and Table 9, as well as in Figure 8, the results of the immunocytochemical examination of SOD1 expression in the single-action model, but also in the combined action model, are presented.

Table 5 shows the result of immunocytochemical localization of SOD1 in MCF-7/WT. In concentrations 0.001–25 µM, 17β-E2 showed an intensity of reaction of (++/+++), and about 50% of the cells were stained. In the case of Cr(VI), a lower percentage of stained cells (18%) was observed, especially for the highest metal concentration. It can be concluded that, compared to 17β-E2, chromium ions induced a lower expression of SOD1 in breast cancer cells (++) in comparison to control (++/+++). Considering the effect of 17β-E2 metabolites, it was noted that 2-MeOE2 induces lower expression of the enzyme as compared to the control (++). The other two metabolites caused a lower expression of SOD1 (++) than the estrogens mentioned above, but comparable to Cr(VI). Table 6, Table 7, Table 8 and Table 9 show the effect of the mixture of estrogen with Cr(VI) on SOD1 expression. The combined protocols, 17β-E2 + Cr(VI), enhanced the intensity of reaction, especially for lower 17β-E2 (0.001 and 0.1 µM). Exposure of cells to 17β-E2 in combination with Cr(VI) (50 µM) resulted in significant expression of SOD1, but mainly at 10 µM 17β-E2. Table 7 shows the combined effect of 2-MeOE2 + Cr(VI) on SOD1 expression. For the lower concentrations of the metabolite (0.001 and 0.1 µM), the intensity of the reaction was high (++/+++), as was the percentage of stained cells (even up to 76%). Although 4-OHE2 produced SOD1 expression (++) in co-exposure with chromium(VI), the percentage of stained cells was highest for the lowest concentration of 4-OHE2 (0.001 µM). The intensity of the reaction was slightly reduced (+++) compared to the control (++/+++) (Table 5). For 16α-OHE1, the intensity of the reaction was mainly in the +/++ range; thus, 16α-OHE1 did not increase SOD1 expression in comparison to control. The degree of SOD1 expression in breast cancer cells under the influence of the tested compounds presented in the Table 5, Table 6, Table 7, Table 8 and Table 9 reflects the microscopic results shown in Figure 8, on which the differential effect of selected concentrations of compounds on the expression of SOD was demonstrated. A fairly high percentage of stained cells was observed after exposure to 17β-E2 at a concentration of 0.1 µM, but also to the combined co-exposure of 17β-E2 with Cr(VI), and 2-MeOE2 with Cr(VI). A low percentage of stained cells was noted after exposure to 16α-OHE1 at a concentration of 0.001 µM and after exposure to the combined effect of this metabolite with Cr(VI) at a concentration of 50. Thus, the effect of the tested compounds on the expression of SOD is very diverse, but with a tendency to lower enzyme expression after exposure to more carcinogenic metabolites, compared to the effect of estradiol alone (See Supplementary Materials).

## 3. Discussion

The ever-growing morbidity of estrogen-dependent breast cancer justifies examining the role of estrogens and some xenoestrogens in this process. This especially concerns metalloestrogens, as interactions between them are still little-known. To our best knowledge, no studies have been carried out to date on the combined action of 17β-estradiol, its metabolites, and Cr(VI) compounds on the formation of DNA strand damage that initiates processes of carcinogenesis [23,24].

An additional interesting aspect was the evaluation of the viability and genotoxicity of 17β-E2 metabolites 4-OHE2, 16α-OHE1, 2-MeOE2, in single and combined action with the metalloestrogen Cr(V). Exposure to high concentrations of estrogens and their metabolites, or to metalloestrogens, may be one of the crucial factors affecting the pathogenesis and progression of estrogen-dependent breast adenocarcinoma [25,26]. Moreover, due to the possible generation of the mechanism of oxidative stress caused by Cr(VI) and the antioxidant properties attributed to estrogens, it seems that they can be expected to act protectively [11,27]. Estrogens may also act pro-oxidatively. Therefore, the problem of combined exposure appears to be complex and justifies researching this direction [9]. Our earlier study demonstrated the existence of 17β-E2, its metabolites, and Cr(VI) interactions in the modulation of oxidative stress (OS) in erythrocytes and mitochondria isolated from the human placenta [10,28]. It has been suggested that 17β-E2 metabolites, for example 4-OHE2, may be carcinogenic in comparison to 17β-E2 or that 2-methoxy derivatives may have strong antioxidant and anticancer properties [29]. Due to the fact that the direction and dynamics of viability changes in both breast cancer lines were similar, further experiments were performed on the MCF-7/WT line [30,31]. The effect of compounds on SOD1 expression was assessed to elucidate the possible role of oxidative stress.

The first question we asked ourselves was how do particular compounds influence cell viability? The trend of changes in both tested lines was similar. A more substantial cytotoxic effect, compared to 17β-E2, was observed for 2-MeOE2, which is consistent with studies conducted by Nair et al. [32]. The authors showed that 2-MeOE2 (1.0 μM) inhibits the viability of MCF-7 cells by as much as 85%. While in our study 4-OHE2 almost completely inhibited MCF-7/WT viability, Chen et al. [33] demonstrated the cytotoxic activity of 4-OHE2 on MCF-10A cells (line of normal mammary gland epithelium), where their proliferation was inhibited in 60%. Fussell et al. [34] indicated the cytotoxic effects of 4-OHE2 and pointed out that catechol metabolites of endogenous estrogens undergo redox cycling in breast epithelial cells, resulting in ROS production. For Cr(VI), a strong cytotoxic effect and ultimately damaged MCF-7/WT were observed in our study. Wei et al. [35] revealed that Cr(VI) penetrated MCF-7 cells more significantly than Cr(III), and its concentration in the culture medium increased exponentially with Cr(VI) concentration. Martin et al. [17] showed the ability of Cr(VI) to activate ERα in cell line-MCF-7. A similar effect of estrogens and xenoestrogens 17β-E2 caused a statistically significant decrease in the viability of the MDA-MB-175-VII cells, while 2-MeOE2 caused an inhibition of viability, similar to 4-OHE2. Summarizing the first stage, the viability of MCF-7/WT and MDA-MB-175-VII cells is lowered most by Cr(VI) and least by 17β-E2.

The second question was how do estrogens, in combined action, with Cr(VI) influence breast cell viability? To our knowledge, there are no data on this topic. For 17β-E2, we observed protection against the cytotoxicity of Cr(VI). The literature describes the use of 17β-E2 for the treatment of breast cancer in postmenopausal women [19]. Evaluating the effect of 2-MeOE2 with Cr(VI), we observed decreased MCF-7/WT viability. Some authors observed that the antitumor effect of 2-MeOE2 is only dependent on binding to the ER to a minimal degree, whereas its action is mainly dictated by damage to the cytoskeleton structure and by blocking SOD activity [17]. In studies on the MDA-MB-175-VII line, the protective effect of 17β-E2 against Cr(VI) was demonstrated. The trend of changes is different for the combined effect of 2-MeOE2 with Cr(VI) than for 17β-E2 but is common to the MCF-7/WT line. While 4-OHE2 inhibited cell viability, the cumulative effect of 16α-OHE1 with Cr(VI) on the trend of changes was similar to that in the MCF-7/WT line. The direction and dynamics of changes in viability after exposure to 4-OHE2 and 16α-OHE1 are similar for both lines. In conclusion, in both lines mainly 17β-E2 was protective when exposed to Cr(VI).

We asked ourselves how estrogens influence DNA damage. Yared et al. [36] observed DNA strand breaks in MCF-7 cells after 2 h exposure to 17β-E2. Similar results were obtained by other authors, who examined physiological concentrations of estradiol (i.e., 0.5 nM, 1 nM, 2 nM) and pointed to DNA damage [37]. The long-term intake of exogenous estrogens is a crucial factor contributing to the increased risk of breast cancer. The role of their metabolites in inducing this process through the mechanism of genotoxic activity is still not fully understood [38]. Using comet assay, Rajapakse et al. [39] observed increases in the number of single-strand breaks in MCF-7 and MDA-MB-231 breast cancer cells exposed to E2 or 4-OHE2. The assessment of the combined impact of 17β-E2, its metabolites, and Cr(VI) in inducing genotoxicity in breast cancer cells was of great interest to us. Cr(VI) is a carcinogen with proven genotoxicity, but it is still being investigated by scientists [40]. In our study, the highest doses of Cr(VI) caused intensified apoptosis of MCF-7 cells. Similar results were obtained by Cavallo et al. [41] for direct-oxidative DNA damage and apoptosis in the human lung (A549) and bronchial (BEAS-2B) cells exposed to 0.1, 0.5, 1.0, and 10 μM sodium chromate, which indicates the concentration-dependent genotoxicity of Cr(VI). Summarizing the third stage genotoxicity evaluation, Cr(VI) caused the most intensive apoptosis, while 17β-E2 caused the lowest. Of course, the phenomenon of apoptosis is a very complex mechanism in which various factors and enzymes from the caspase group, in addition to initiating and executive caspases, are involved [42].

The fourth question was how do estrogens influence genotoxicity in combined action with Cr(VI)? Low doses of Cr(VI) and 17β-E2 caused interaction with a high percentage of intermediate damage; however, high apoptosis was achieved, indicating synergism. As the literature data show, DNA adducts may be formed after the metabolization of a steroid [43]. Since 2-MeOE2 is also an inhibitor of microtubule formation, it induces aneuploidy and mammalian cell mutation, as demonstrated by in vitro studies on the SHE cell line (Syrian hamster embryo fibroblast) exposed to 2-MeOE2 [44,45]. Studies by Khoei et al. [46] also showed the participation of 2-MeOE2 (250 μM) in the formation of DNA strand breaks on the U87MG cell line (human glioblastoma). Saczko et al. [21] revealed the genotoxicity of 2-MeOE2 on the MCF-7 and OvBH-1 cell lines (ovarian clear cell carcinoma cells). The combined action of 2-MeOE2 and Cr(VI) used in our experiments resulted in the intensification of apoptosis. For combined action of 2-MeOE2 and Cr(VI), apoptosis was as high as when exposed to Cr(VI) alone. The larger DNA damage caused by 2-MeOE2 can probably be explained by the multidirectional mechanism of action of 2-MeOE2, which, in combined action, increased the number of DNA damage caused by Cr(VI) in low doses.

SOD1, a copper–zinc (Cu/Zn SOD) isoform, is a variant located in the cellular cyto-plasm and mitochondrial inter-membrane space. As an antioxidant enzyme, it is overexpressed in cancers, and its activity may be essential to maintaining cellular ROS under the critical threshold [47]. SOD1 is overexpressed in most malignant breast cancer cells (e.g., MCF-7). Conversely, it is reduced in non-cancer cells, e.g., on the MCF-10A cell line [48]. Our observations found a higher expression of SOD1 in MCF-7/WT cells exposed to 17β-E2. This dependence was not noticed for 16α-OHE1 and 4-OHE2 or for 2-MeOE2, which decreased SOD1 expression on its own. Xenobiotic interactions affect SOD1 expression in comparison to single action. The highest intensity was observed for the action of Cr(VI)+17β-E2 and for the Cr(VI)-+2-MeOE2 interaction. Our studies indicated that SOD1 was present in the cell lines tested, but SOD1 expression is often diminished or enlarged relative to the controls not exposed to estrogens or Cr(VI). Increased activity of SOD1 in breast cancer cells may contribute to the increased resistance of breast cancer cells to oxidative stress [49]. According to a report by Glasauer, the inhibition of SOD1 leads to a decrease in the concentration of antiapoptotic factors and to the higher apoptosis of lung cancer cells [50]. These results indicate that this antioxidant enzyme may play a key role in the survival mechanisms of tumor cells associated with oxidative stress. Therefore, SOD1 is a potential target for anti-cancer therapies, and its inhibitors may find application in anticancer therapy. Our research on SOD1 expression requires continuation and determination of the remaining antioxidant enzymes to draw more specific conclusions.

From the point of view of antitumor therapy, 2-MeOE2, as an effect of chemotherapy, causes an increase in the number of apoptotic cells while reducing the number of necrotic cells. This is the desired phenomenon during chemotherapy because it limits the formation of a large amount of necrotic tissue at the site of tumor lysis, which is dangerous for patients [24]. There are some scientific studies confirming the presence of 4-OHE2 adducts in breast cancer [51,52]. Although the literature data on the genotoxicity of 4-OHE2 are sparse, it has been indicated that long-term exposure to 4-OHE2 may be one of the most important risk factors for breast cancer [53]. According to research by Rajapakse et al. [39] on the MCF-7 cell line and Zahid et al. [54] in MCF-10A cells, 4-OHE2 leads to increased DNA damage. These studies proved that catechol metabolites can become chemical carcinogens in reaction with DNA. Studies on the SHE cell line have shown that 4-OHE2 also causes structural changes in chromosomes as well as aneuploid changes [45]. In our observations, simultaneous exposure to Cr(VI) with 4-OHE2 caused a significant increase in indirect damage and an increase in apoptosis. This combined effect was similar to the effect of 2-MeOE2, where almost complete apoptosis was noted, while different to the combined effect of Cr(VI) and 17β-E2, where antagonism was observed. The literature data on the genotoxic effect of 16α-OHE1 are scarce. Only Tsutsui et al. [55] observed genotoxicity of 16α-OHE1 in the form of aneuploidy on the SHE cell line. The genotoxicity of estrogens is closely related to their hydroxylation, which often determines steroids’ toxicity. Summarizing the fourth stage of the study, we revealed that co-exposure with Cr(VI) increases apoptosis, especially in the case of 4-OHE2 and 2-MeOE2. Moreover, high doses of Cr(VI) cause the greatest damage to cells (apoptosis) as well as the greatest cytotoxicity in the MTT test.

Both used cancer lines are estrogen-dependent. Receptors for glucocorticoids, progestogens, and androgens have been identified in these human breast cancer cell lines known to have estrogen receptors, so it may be an excellent in vitro model to study the mechanism of tumor response to estrogens as well as xenoestrogens [56,57]. While 17β-E2 acts through estrogen receptors, the effect of 2-MeOE2 is not estrogen-dependent, which may explain the different responses of the studied estrogen on dependent breast cancer lines. Significant differences obtained between viability and apoptosis have yet to be confirmed in further studies. This preliminary research needs to be continued.

Summarizing very briefly, the viability of MCF-7/WT and MDA-MB-175-VII cells is lowered most by Cr(VI) and least by 17β-E2. In combined action, the protective effect of 17β-E2 against Cr(VI) was demonstrated both in MCF-7/WT and MDA-MB-175-VII. The direction and dynamics of changes in viability after combined exposure to Cr(VI) +4-OHE2 and Cr(VI) +16α OHE1 are similar for both lines. Evaluating the effect of 2-MeOE2 with Cr(VI), we observed viability to be decreased in MCF-7/WT and MDA-MB-175-VII. Cr(VI) alone induced significant cell apoptosis. Cr(VI) +17β-E2 caused an interaction with a high percentage of intermediate damage. The combined action of 2-MeOE2 and Cr(VI) resulted in intensification of the apoptosis. High SOD1 expression was found in MCF-7/WT cells exposed to 17β-E2.

## 4. Materials and Methods

### 4.1. Cell Culture

The study was performed on estrogen-dependent breast cancer cell lines MCF-7/WT and MDA-MB-175-VII. Estrogen-dependent breast adenoma cell line MCF-/W7 was purchased from CLS Cell Lines Service GmbH, Eppelheim, Germany (Product number: 300273). The MDA-MB-175 cell line (ATCC: HTB-25) was obtained from ATCC. This cell line was derived from the Department of Tumor Biology of the Wroclaw Medical University, Faculty of Pharmacy, in Wroclaw, Poland. The cultures were maintained at 37 °C under high humidity in the Steri-Cult^®^ Automated CO_2_ Incubator (Steri-Cult, Thermo Scientific, Alab, Poland). The MCF-7/WT line was grown in DMEM (Dulbecco’s Modified Eagle’s Medium) with a 4500 mg/L concentration of glucose (Sigma-Aldrich, Poznań, Poland) supplemented with 10% fetal bovine serum (FBS, Sigma-Aldrich, Poznań, Poland) and 1% antibiotic solution which included 10,000 units of penicillin and 10 mg of streptomycin/mL (Sigma, Saint Louis, USA). The MDA-MB-175-VII cell line was first isolated in 1973 from a 56-year-old black woman with a pleural effusion who had metastatic disease. This epithelial cell was cultured in Leibovitz’s L-15 culture medium, which includes 2 mM L-glutamine and 10% fetal bovine serum (FBS).

### 4.2. Compounds

Five compounds were applied in the study: 17β-E2, 2-MeOE2, 4-OHE2, 16α-OHE1, and potassium chromate(VI)—K2CrO4, as a source of hexavalent chromium—Cr(IV) (Table 10). The compounds were used at starting concentrations of 50 mM for 17β-E2 and 10 mM for its metabolites (96% ethanol solutions) and hexavalent chromium at 50 mM (aqueous solutions). Before conducting the experiments, all stock solutions of the examined compounds were diluted in the culture medium (DMEM) to obtain the appropriate concentrations. The following concentrations (nM and µM) were used for the estrogen examination: 0.1, 1.0, 10. and 100 nM and 0.5, 1.0, 5.0, 10, 25, and 50 µM. In order to evaluate the effect of Cr(VI), the following 15 concentrations of K2CrO4 were used: 0.01, 0.02, 0.05, 0.1, 0.2, 0.5, 1.0, 2.0, 5.0, 10.0, 20.0, 40.0, 50.0, 100.0, and 200.0 µM. The study on the second cell line, MDA-MB-175-VII, was performed with concentrations of 0.001, 0.1, 0.5, 10, and 25 µM (estrogens) and 0.1, 1, 5, 10, and 50 µM-Cr(VI).

### 4.3. MTT Assay

**Figure 9:**
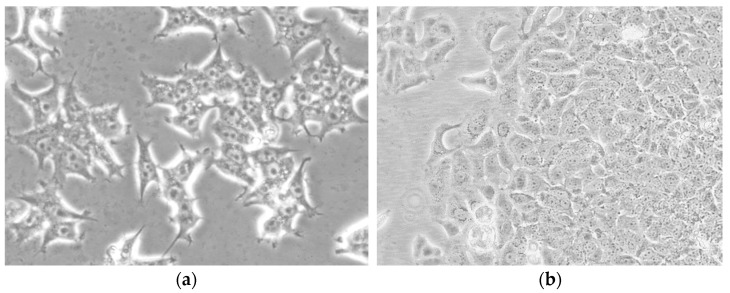
Sample micrograph presenting cell line cultures: MCF-7/WT (**a**) and MDA-MB-175-VII (**b**) (Magnification scale 20×).

The viability of the examined compounds was assessed by an MTT test performed according to a standard procedure (Sigma-Aldrich, Poznań, Poland) in 96 well plates (Nunc, NunclonTM Surface, Biokom, Poland). Cells in DMEM (MCF-7/WT) and in Leibovitz’s L-15 culture medium (MDA-MB-175-VII) were seeded into each well at the density of 104 cells/well. The plates were incubated at 37 °C and 5% CO_2_ for 24 h to allow for cell attachment. Sample micrograph presenting cell line culture MCF-7/WT (Figure 9a) and MDA-MB-175-VII (Figure 9bAfter 24 h, the medium was removed and replaced with 200 µL solutions of examined estrogens or Cr(VI). Varying concentrations of estrogens and Cr(VI) or a combination of compounds were added to cells. For negative control, only a complete growth medium was added into wells seeded with cells.

First, the effect on the viability of a single compound was conducted. For this purpose, cells were incubated with estrogens 17β-E2, 2-MeOE2, 4-OHE2, and 16α-OHE1 or Cr(VI) independently in concentrations indicated in the “Compounds” section above. The experiment was performed in triplicate to validate the data obtained. The plates were incubated for 72 h. After that, 20 μL of MTT (3-(4,5-dimethylthiazol-2-yl)-2,5-diphenyltetrazolium bromide) (5 mg/mL) was added into each well for a final concentration of 0.5 mg/mL and incubated for 4 h. To release formazan crystals accumulated inside the cells and dissolve them, a lysis buffer consisting of 25 mL isopropanol and 100 μL of 38% hydrochloric acid solution was used. The absorbance was measured at Perkin Elmer Enspire Plate Reader (Perkin Elmer, Waltham, MA, USA) at a wavelength of 570 nm. The results were presented as viability (% of control). Cell viability was expressed as the percentage of viable cells in relation to untreated control cells.

**Figure 10:**
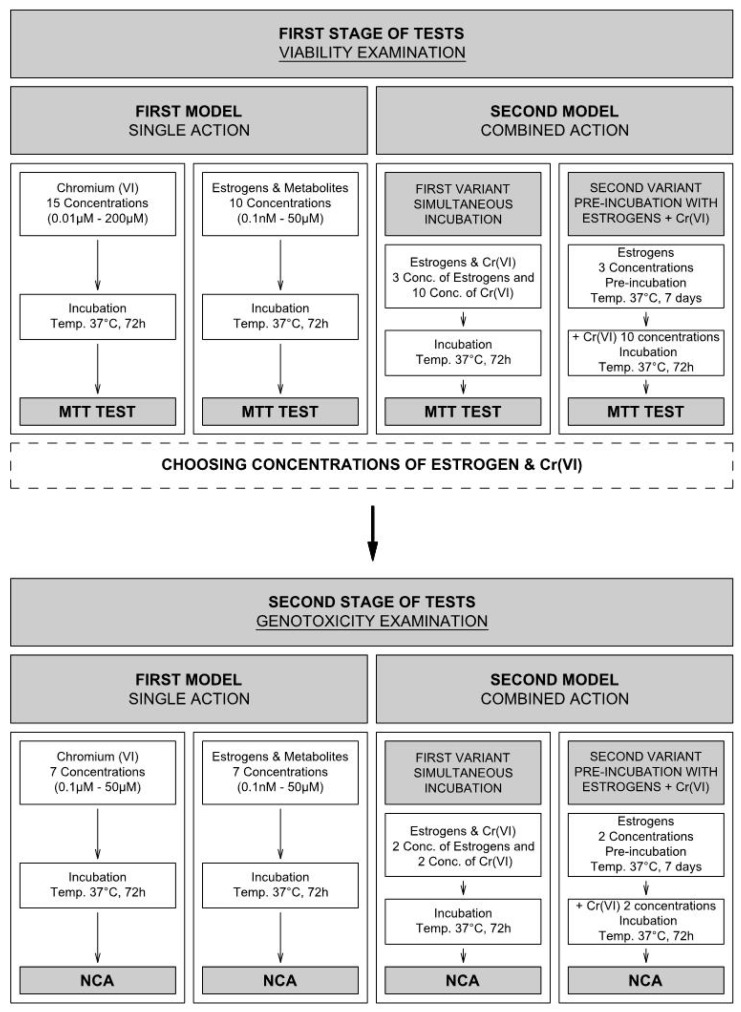
Study design for testing the viability (MTT) and genotoxicity (NCA) of estradiol and its metabolites with Cr(VI) in single and combined action.

Secondly, the effect on the viability of combined action of estrogens–Cr(VI) was evaluated. In simultaneous incubation, 200 μL of a mixture of compounds were incubated with cells for 72 h. Moreover, a pre-incubation model was followed, where cells were initially incubated with the appropriate estrogen (7 days) and then with Cr(VI) (72 h) (Figure 10). The compound concentrations, based on the individual study, were chosen at this stage: for 17β-E2 and 16α-OHE1 1.0 nM, 10.0 µM, and 25 µM; for 2-MeOE2 and 4-OHE2 1.0 nM, 0.5 µM, and 10.0 µM; while Cr(VI) for the MCF-7/WT line was used in ten concentrations: 0.1, 0.2, 0.5, 1.0, 5.0, 10.0, 20.0, 40.0, and 50.0 µM, and for the MDA-MB-175-VII line doses were 0.1, 1, 5, 10, and 50 µM. In the pre-incubation model, estrogen was added to the culture medium, and the cells were incubated in the culture flask for 7 days (with a million cells per flask). After this time, cells were trypsynized and seeded in 104 cell/well assay plates and incubated for 24 h for the cells to stick to the wells. K_2_CrO_4_ was then added to the culture medium in appropriate concentrations (72 h incubation). Next, the MTT test was performed according to standard procedure (Merck, Poland-Sigma-Aldrich)). All experiments were performed in triplicate to validate the data obtained.

### 4.4. Neutral Comet Assay (NCA)

Detection of DNA fragmentation associated with apoptosis or the intermediate damage neutral comet assay method described by Collins was used [58]. Briefly, after exposure to the examined compounds, MCF-7/WT cells (104 cells/well) were subjected to a trypsinization process. In the next step, 2 × 104 cells were washed with PBS chilled to 4 °C, mixed with low temperature melting agarose at a ratio of 1:10 (*v*/*v*), and spread on a slide glass. Slides were submerged in precooled lysis buffer (2.5 M NaCl, 100 mM EDTA, pH 10, 10 mM Tris base, and 1% Triton X-100) at 4 °C for 60 min. After lysis and rinsing, slides were equilibrated in TBE solution (40 mM Tris/boric acid, 2 mM EDTA, pH 8.3) and electrophoresed at 1.0 V/cm for 20 min, and then silver staining was performed. For scoring the comet pattern, 100–200 nuclei were counted from each slide. The ranking of apoptotic comets by the method developed by Collins was performed. The Olympus BX 51 light microscope (Olympus, Japan) was used to count comets, while the Color View IIIU camera and computer with Cell ^ F software were used to take pictures and measure the dimensions of the comets. The results are presented as frequencies of comets in each class, calculated from the ratio of the length of the comet’s tail to the diameter of the cell nucleus (G0—no damage; G1–2—intermediate damage; G3—apoptosis) [59,60]. Under neutral conditions, we could mainly detect DNA double-strand breaks considered by our estimations to be suitable for detecting apoptosis. Using this method, we evaluated the percentage of apoptotic cells.

The genotoxic activity of individual estrogens and Cr(VI) on MCF-7/WT cells was performed according to the scheme (see in detail in Figure 10). In the examination of individual genotoxic activity (first model), solutions of Cr(VI) were used in the following concentrations: 0.1, 0.5, 1.0, 5.0, 10.0, 20.0, and 50.0 μM, while the genotoxicity of individual estrogens was examined in concentrations: 0.1 nM, 1.0 nM, 0.1 μM, 1.0 μM, 5.0 μM, 25.0 μM, and 50.0 μM, selected on the basis of the viability results. Then the Neutral Comet Assay was conducted as was described in detail above. The genotoxicity assay of the combined effect of estrogens–Cr(VI) was conducted in two variants (Figure 10). Based on the genotoxicity of single compounds, two doses of each estrogen, 1.0 nM and 25 µM, and two concentrations of Cr(VI), 0.1 µM and 20.0 µM, were selected. Then, the NCA was conducted and is described in detail above.

### 4.5. SOD1 Estimation: Immunocytochemical Staining

Additionally, the expression of SOD1 was investigated in MCF-7/WT cells. This study determines the expression of SOD1 in breast cancer cells exposed to the single compounds and mixture of estrogens and Cr(VI) similar to the viability test. Cells were plated on 10-well slides (Thermo Scientific, Waltham, MA, USA) and incubated for 24 h. The slides were then rinsed with PBS and fixed with 4% paraformaldehyde. The next step was to perform an immunocytochemical test with the Expose Mouse and Rabbit Specific HRP/DAB Detection IHC kit (Abcam, Waltham, MA, USA, ab80436). The kit contained reagents: Mouse Determination Reagent, HRP Conjugate, DAB Substrate, DAB Chromogen, and Hydrogen Peroxide Block. After washing with PBS (3 × 5 min), peroxidase activity was blocked by 30 min incubation with 1% H2O2; then, samples were permeabilized by incubation with 1% Triton X-100 (Merck, Poland-Sigma-Aldrich)) in PBS (LabEmpire, Rzeszów, Poland). Cells were then incubated with the selected antibodies overnight at 4 °C. A primary antibody was used: anti-SOD1 rabbit polyclonal antibody (orb39428, Biorbyt, Cambridge, UK). Cells were incubated with a secondary horseradish peroxidase (HRP) conjugated antibody. The samples were then incubated with a mixture of diaminobenzidine-H2O2 to show the HRP marker and were counterstained with hematoxylin (Roth, Poland) for 3 min. After dehydration in a gradient of ethanol (Chempur, Rzeszów, Poland) and xylene (Chempur, Piekary Śląskie, Poland), the microscope slides were covered with DPX (Aqua-Med ZpamKolasa, Łódź, Poland). A vertical microscope (Olympus BX53, Warszawa, Poland) was used for sampling. The number of stained cells was determined by counting 100 cells in 3 randomly selected fields. First, the staining of the cells was tested. The percentage of stained cells was shown in the table, after which the intensity of the staining was estimated. The intensity of the immunohistochemical staining was assessed as (-) negative (no reaction), (+) weak, (++) moderate, and (+++) strong.

### 4.6. Schematic Model for Viability and Genotoxicity Testing

To better visualize a large number of viability analyses, followed by genotoxicity, we prepared a test pattern (Figure 10). The viability and genotoxic effects, assigned as first and second stages of tests, were induced. The single action of compounds (first model) and the combined effect of estrogens with Cr(VI) (second model) on cells were examined. The combined effect was examined in two variants: simultaneous incubation of examined compounds (first variant) and with pre-incubation (second variant) with estrogen (7 days) and then incubation with Cr(VI) (72 h).

### 4.7. Statistical Analysis

Three independent experiments were performed for all assays. Data analysis was performed using Microsoft Excel and GraphPad Prism 7. Results with *p* < 0.05 were considered to be statistically significant. One-way analysis of variance (ANOVA) was used for the significance testing. All results in the graphs are presented as mean ± SEM.

## 5. Conclusions

In conclusion, the study performed in MCF-7/WT and MDA-MB-175-VII cells has established that interactions between Cr(VI) and 17β-E2, 2-MeOE2, 4-OHE2, and 16α-OHE1 can change the viability of the examined breast cancer lines. The presented study has demonstrated the protective effect of 17β-E2 against Cr(VI)-induced cytotoxicity. The direction and dynamics of changes in the viability are consistent for both lines. The study also provided evidence indicating the potential involvement of the studied estrogens and Cr(VI) in DNA damage in estrogen-dependent breast cancer cells. Exposure to cytotoxic compounds induced a various response of the antioxidant system measured by SOD expression, which can be explained by differences in their accumulation and biotransformation in tumor cells. Our research showed that interactions between estrogens and metalloestrogens may play a role in the dysfunction of hormone-dependent breast cancer cells, but, given the preliminary nature of this study, further research is needed in this area.

## Figures and Tables

**Table 1:** IC50 for 17β-E2 and Cr(VI), for 2-MeOE2 and Cr(VI), for 4-OHE2 and Cr(VI), and for 16α-OHE1 and Cr(VI) after simultaneously effect on MCF-7/WT, measured by MTT (expressed in µM).

IC 50 for Examined Compounds			
Cr(VI)	0.001 µM 17β-E2	10 µM 17β-E2	25 µM 17β-E2
5.2251	4.2423	2.1506	0.4278
Cr(VI)	0.001 µM2-MeOE2	0.5 µM 2-MeOE2	10 µM 2-MeOE2
5.2251	6.4528	3.2515	2.1813
Cr(VI)	0.001 µM 4-OHE2	0.5 µM 4-OHE2	10 µM 4-OHE2
5.2251	3.2589	4.5478	6.8237
Cr(VI)	0.001 µM 16α-OHE1	10 µM16α-OHE1	25 µM16α-OHE1
5.2251	4.2465	3.5783	6.8957

**Table 2:** IC50 for 17β-E2 and Cr(VI), for 2-MeOE2 and Cr(VI), for 4-OHE2 and Cr(VI), and for 16α-OHE1 and Cr(VI) after 7 days pre-incubation with estrogen on MCF-7/WT, measured by MTT (expressed in µM).

IC 50 for Examined Compounds				
17β-E2	Cr(VI)	7 days pre-inc. 0.001 µM 17β-E2	7 days pre-inc. 10 µM 17β-E2	7 days pre-inc. 25 µM 17β-E2
	5.2251	6.6895	4.9685	12.3467
2-MeOE2	Cr(VI)	7 days pre-inc. 0.001 µM 2-MeOE2	7 days pre-inc. 0.5 µM 2-MeOE2	days pre-inc. 10 µM 2-MeOE2
	5.2251	4.2584	6.9854	0.2584
4-OHE2	Cr(VI)	7 days pre-inc. 0.001 µM 4-OHE2	7 days pre-inc. 0.5 µM 4-OHE2	7 days pre-inc. 10 µM 4-OHE2
	5.2251	3.8255	4.2857	7.2824
16α-OHE1	Cr(VI)	7 days pre-inc. 0.001µM 16α-OHE1	7 days pre-inc. 10 µM 16α-OHE1	7 days pre-inc. 25 µM16α-OHE1
	5.2251	7.2867	9.5857	2.9487

**Table 3:** IC50 for 17β-E2 and Cr(VI) for 2-MeOE2 and Cr(VI), for 4-OHE2 and Cr(VI), and for 16α-OHE1 and Cr(VI) after simultaneously effect on MDA-MB-175-VII cell line, measured by MTT (expressed in µM).

IC 50 for Examined Compounds			
Cr(VI)	0.001 µM 17β-E2	10 µM 17β-E2	25 µM 17β-E2
5.2251	3.9223	2.1789	0.6267
Cr(VI)	0.001 µM2-MeOE2	0.5 µM 2-MeOE2	10 µM 2-MeOE2
5.2251	5.8728	3.9875	1.9874
Cr(VI)	0.001 µM 4-OHE2	0.5 µM 4-OHE2	10 µM 4-OHE2
5.2251	2.9749	3.7838	7.4537
Cr(VI)	0.001 µM 16α-OHE1	10 µM16α-OHE1	25 µM16α-OHE1
5.2251	3.9865	2.9783	6.0743

**Table 4:** IC50 for 17β-E2 and Cr(VI), for 2-MeOE2 and Cr(VI), for 4-OHE2 and Cr(VI), and for 16α-OHE1 and Cr(VI) after 7 days pre-incubation with appropriate estrogen on MDA-MB-175-VII cell line, measured by MTT (expressed in µM).

IC 50 for Examined Compounds				
17β-E2	Cr(VI)	7 days pre-inc. 0.001 µM 17β-E2	7 days pre-inc. 10 µM 17β-E2	7 days pre-inc. 25 µM 17β-E2
	4.5671	5.9795	5.3485	10.8767
2-MeOE2	Cr(VI)	7 days pre-inc. 0.001 µM 2-MeOE2	7 days pre-inc. 0.5 µM 2-MeOE2	7 days pre-inc. 10 µM 2-MeOE2
	6.1151	3.9985	7.1540	0.1875
4-OHE2	Cr(VI)	7 days pre-inc. 0.001 µM 4-OHE2	7 days pre-inc. 0.5 µM 4-OHE2	7 days pre-inc. 10 µM 4-OHE2
	5.5461	4.1563	4.7854	6.8374
16α-OHE1	Cr(VI)	7 days pre-inc. 0.001µM 16α-OHE1	7 days pre-inc. 10 µM16α-OHE1	7 days pre-inc. 25 µM16α-OHE1
	4.8451	6.1127	7.9987	2.3567

**Table 5:** Positive grading quantification of immunocytochemical staining of SOD1 in the MCF-7/WT line under the influence of compounds. The number of stained cells was determined by counting 100 cells in three randomly selected fields. The intensity of the immunohistochemical staining was assessed as (-) negative (no reaction), (+) weak, (++) moderate, and (+++) strong.

Compound	% of Stained Cells	Intensity of the Reaction
17β-E2 [µM]		
0.001	42%	+++
0.1	59%	++
10	56%	++/+++
25	45%	++/+++
Cr(VI)[µM]		
0.1	29%	+/++
1	32%	++
5	22%	++
10	37%	++
50	18%	++
2ME_2_ [µM]		
0.001	38%	++
0.1	42%	++
0.5	39%	++
10	27%	++
4-OH [µM]		
0.001	25%	++
0.5	18%	++
10	14%	++
25	34%	++/+++
16α-OHE_1_ [µM]		
0.001	28%	++
1	32%	++
10	19%	++
25	35%	++
Control	43%	++/+++

**Table 6:** Positive grading quantification of immunocytochemical staining of SOD1 in the MCF-7/WT line under the influence of 17β-E2 with Cr(VI). The number of stained cells was determined by counting 100 cells in three randomly selected fields. The intensity of the immunohistochemical staining was assessed as (-) negative (no reaction), (+) weak, (++) moderate, and (+++) strong.

Cr(VI) [µM]	% of Stained Cells	Intensity of the Reaction	Cr(VI) [µM]	% of Stained Cells	Intensity of the Reaction
17β-E2 0.001 µM			17β-E2 10 µM		
0.1	21%	++	0.1	49%	++
1	39%	+/++	1	41%	++
5	28%	++	5	38%	++
10	27%	++/+++	10	46%	++/+++
50	52%	++/+++	50	68%	+++
17β-E2 0.1 µM			17β-E2 25 µM		
0.1	32%	++/+++	0.1	47%	++
1	38%	++/+++	1	38%	++
5	41%	++	5	39%	++
10	49%	++	10	37%	++
50	43%	++	50	24%	++
Control	43%	++/+++	Control	43%	++/+++

**Table 7:** Positive grading quantification of immunocytochemical staining of SOD1 in the MCF-7/WT line under the influence of 2-MeOE2 with Cr(VI). The number of stained cells was determined by counting 100 cells in three randomly selected fields. The intensity of the immunohistochemical staining was assessed as (-) negative (no reaction), (+) weak, (++) moderate, and (+++) strong.

Cr(VI) [µM]	% of Stained Cells	Intensity of the Reaction	Cr(VI) [µM]	% of Stained Cells	Intensity of the Reaction
2-MeOE2 0.001 µM			2-MeOE2 0.5 µM		
0.1	42%	++	0.1	54%	++
1	56%	++	1	44%	++
5	61%	++/+++	5	27%	++
10	70%	++/+++	10	42%	++
50	76%	++/+++	50	51%	++
2-MeOE2 0.1 µM			2-MeOE2 10 µM		
0.1	60%	++	0.1	45%	++
1	50%	++	1	43%	++
5	45%	++	5	35%	+
10	30%	++/+++	10	43%	++
50	50%	++/+++	50	59%	++
Control	43%	++/+++	Control	43%	++/+++

**Table 8:** Positive grading quantification of immunocytochemical staining of SOD1 in the MCF-7/WT line under the influence of 4-OHE2 with Cr(VI). The number of stained cells was determined by counting 100 cells in three randomly selected fields. The intensity of the immunohistochemical staining was assessed as (-) negative (no reaction), (+) weak, (++) moderate, and (+++) strong.

Cr(VI) [µM]	% of Stained Cells	Intensity of the Reaction	Cr(VI) [µM]	% of Stained Cells	Intensity of the Reaction
4-OHE2 0.001 µM			4-OHE2 0.5 µM		
0.1	42%	+++	0.1	22%	++
1	36%	++	1	32%	++
5	47%	++	5	17%	++
10	40%	++	10	22%	+/++
50	56%	+++	50	21%	++
4-OHE2 10 µM			4-OHE2 25 µM		
0.1	25%	++	0.1	25%	++
1	16%	++	1	34%	++
5	27%	++	5	21%	+
10	23%	++	10	29%	+/++
50	37%	++	50	28%	++
Control	43%	++/+++	Control	43%	++/+++

**Table 9:** Positive grading quantification of immunocytochemical staining of SOD1 in the MCF-7/WT line under the influence of 16α-OHE1 with Cr(VI). The number of stained cells was determined by counting 100 cells in three randomly selected fields. The intensity of the immunohistochemical staining was assessed as (-) negative (no reaction), (+) weak, (++) moderate, and (+++) strong.

Cr(VI) [µM]	% of Stained Cells	Intensity of the Reaction	Cr(VI)_2_ [µM]	% of Stained Cells	Intensity of the Reaction
16α-OHE_1_ 0.001 µM			16α-OHE_1_ 10 µM		
0.1	35%	++	0.1	43%	++
1	29%	++	1	44%	++
5	45%	++	5	38%	++
10	28%	++	10	56%	+
50	35%	+	50	34%	+
16α-OHE_1_ 1 µM			16α-OHE_1_ 25 µM		
0.1	50%	++	0.1	45%	++
1	42%	++	1	33%	++
5	39%	++	5	45%	++
10	49%	+/++	10	53%	++
50	38%	++	50	59%	++
Control	43%	++/+++	Control	43%	++/+++

**Table 10:** The list of tested compounds.

Name of Compound	Structure and Manufacturer’s Name	Molecular Mass of Compound
17β-E2	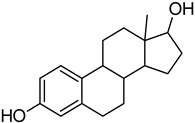 SIGMA-ALDRICH	272.38 g/mol
2-MeOE2	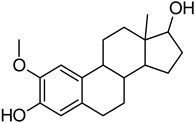 SIGMA-ALDRICH	302.41 g/mol
4-OHE2	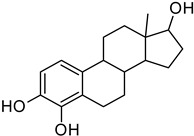 SIGMA-ALDRICH	288.38 g/mol
16α-OHE1	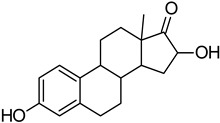 STERALOIDS	286.40 g/mol
potassium chromate (VI)	K2CrO4 SIGMA-ALDRICH	194.19 g/mol

## Data Availability

The data presented in this work are available in the article and supplementary materials.

## Supplementary Materials

The following supporting information can be downloaded at: https://www.mdpi.com/article/10.3390/molecules28062752/s1. Table S1. The apoptosis, indirect damage, and undamaged cells (neutral comet assay) in MCF-7/WT cell line after exposure to single compounds: 17β-E2, 2-MeOE2, 4-OHE2, and 16α-OHE1. Table S2. The apoptosis, indirect damage, and undamaged cells (neutral comet assay) in MCF-7/WT cell line after exposure to Cr(VI). Table S3. The apoptosis, indirect damage, and undamaged cells (neutral comet assay) in MCF-7/WT cell line after exposure to simultaneous effect of estrogens with Cr(VI) (Int) or after pre-incubation with estrogen (P-I) and next Cr(VI) exposure.

## Sample Availability

Samples of the compounds are not available from the authors.
