# Supplementary material for: Effect of Interaction between Chromium(VI) with 17β-Estradiol and Its Metabolites on Breast Cancer Cell Lines MCF-7/WT and MDA-MB-175-VII: Preliminary Study
Source: Molecules. 2023 Mar 18;28(6):2752. doi: 10.3390/molecules28062752 (PMC10052759; doi:10.3390/molecules28062752)
Supplement: Supplementary file 1 [file molecules-28-02752-s001.zip › molecules-2282360-supplementary.pdf]

Table S1. The apoptosis, indirect damage and undamaged cells (neutral comet assay) in MCF-7/WT cell line after exposure to single compounds: 17β-E2, 2-MeOE2, 4-OHE2 and 16α-OHE1.

**17β-E2**								
	control cells	0.1 nM	1 nM	0.1 uM	1 uM	5 uM	25 uM	50 uM
not damage(%)	98	85	80	68	49	47	42	32
intermediate damage(%)	2	13	27	28	39	43	42	50
apoptosis(%)	0	2	3	4	12	10	16	18
**2-MeOE2**								
not damage(%)	98	99	99	95	48	30	24	12
intermediate damage(%)	2	1	1	5	40	42	42	56
apoptosis(%)	0	0	0	0	12	28	34	32
**4-OHE2**								
not damage(%)	98	99	79	52	48	44	23	20
intermediate damage(%)	2	1	21	33	33	40	47	52
apoptosis(%)	0	0	0	15	19	16	30	28
**16α-OHE1**								
not damage(%)	98	96	88	80	36	28	20	22
intermediate damage(%)	2	4	8	8	38	30	52	42
apoptosis(%)	0	0	4	12	26	32	28	36

Table S2. The apoptosis, indirect damage and undamaged cells (neutral comet assay) in MCF-7/WT cell line after exposure to Cr(VI).

**Chromium(VI)**								
	control cells	0.1 uM	0.5 uM	1 uM	5 uM	10 uM	20 uM	50 uM
not damage(%)	98	63	53	55	36	32	0	0
intermediate damage(%)	2	30	39	37	49	50	8	0
apoptosis(%)	0	7	8	8	15	18	92	100

Table S3. The apoptosis, indirect damage and undamaged cells (neutral comet assay) in MCF-7/WT cell line after exposure to simultaneous effect of estrogens with Cr(VI) (Int) or after pre-incubation with estrogen (P-I) and next Cr(VI) exposure.

**Combined effect of 17β-E2 with Cr(VI)**									
	17β-E2 1 nM	Cr(VI) 0.1 µM	Int	P-I		17β-E2 25 µM	Cr(VI) 20 µM	Int	P-I
not damage(%)	80	63	20	0		42	0	15	10
intermediate damage(%)	27	30	60	20		42	8	53	60
apoptosis(%)	3	7	20	80		16	92	32	30
**Combined effect of 2-MeOE2 with Cr(VI)**									
	2-MeOE2 1nM	Cr(VI) 0.1 µM	Int	P-I		2-MeOE2 25 µM	Cr(VI) 20 µM	Int	P-I
not damage(%)	99	63	20	15		34	0	0	0
intermediate damage(%)	1	30	35	30		42	8	12	5
apoptosis(%)	0	7	45	55		30	92	88	95
**Combined effect of 4-OHE2 with Cr(VI)**									
	4-OHE2 1 nM	Cr(VI) 0.1 µM	Int	P-I		4-OHE2 25 µM	Cr(VI) 20 µM	Int	P-I
not damage(%)	79	63	0	0		23	0	0	0
intermediate damage(%)	21	30	28	25		47	8	2	4
apoptosis(%)	0	7	72	75		30	92	98	96
**Combined effect of 16α-OHE1 with Cr(VI)**									
	16α-OHE1 1 nM	Cr(VI) 0.1 µM	Int	P-I		16α-OHE1 25 µM	Cr(VI) 20 µM	Int	P-I
not damage(%)	88	63	30	27		20	0	40	48
intermediate damage(%)	8	30	20	28		52	8	23	22
apoptosis(%)	4	7	50	45		28	92	27	30
